# Self-compassion, optimism and shame in childhood trauma among 18-25 years old individuals

**DOI:** 10.1192/j.eurpsy.2021.1927

**Published:** 2021-08-13

**Authors:** G. Dalkılınç, S. Gültekin, B. Güloğlu

**Affiliations:** Psychological Counseling, Bahcesehir University, Istanbul, Turkey

**Keywords:** Shame, Optimism, childhood-trauma, Self-compassion

## Abstract

**Introduction:**

Childhood trauma has a negative impact on mental health of individuals. Self-compassion involves being open to painful and troubling feelings, approaching them in a caring and loving way, accepting negative experiences as a part of human life. Optimism is an individual’s belief that everything will be better in the future despite the difficulties and obstacles of life. Shame is the feeling that occurs when an inadequacy or inappropriate behavior is noticed.

**Objectives:**

The aim of this study was to investigate the effects of childhood trauma on self-compassion, optimism, and shame.

**Methods:**

Childhood Trauma Scale, Self-Compassion Scale, Life Orientation Test and Shame Scale were administered to 384 individuals (304 Female and 80 Male). Their age range was between 18 and 25, with the mean of 21.26.

**Results:**

The findings of MANOVA indicated that a significant main effect of gender on emotional abuse and sexual abuse however there was no main effect of gender on physical abuse, physical neglect, emotional neglect, and excessive protection. Women were exposed to emotional and sexual abuse more than men. MANOVA that was applied to the scores of CTQ revealed a significant overall main effect of self-compassion and optimism whereas there was no main effect of shame.

**Conclusions:**

While self-compassion and optimism are the protective factors for the traumatized individuals, shame is the risk factor.

**Disclosure:**

No significant relationships.

